# Simultaneous Determination of Selegiline, Desmethylselegiline, R/S-methamphetamine, and R/S-amphetamine on Dried Urine Spots by LC/MS/MS: Application to a Pharmacokinetic Study in Urine

**DOI:** 10.3389/fchem.2019.00248

**Published:** 2019-04-17

**Authors:** Lizhu Chen, Yingjia Yu, Gengli Duan, Xin Wang, Baohua Shen, Ping Xiang

**Affiliations:** ^1^Shanghai Key Laboratory of Forensic Medicine, Department of Forensic Toxicology, Academy of Forensic Science, Shanghai, China; ^2^Department of Pharmaceutical Analysis, School of Pharmacy, Fudan University, Shanghai, China

**Keywords:** urine, chiral analysis, pharmacokinetic, R/S-methamphetamine, R/S-amphetamine, selegiline

## Abstract

**Objective:** Chiral analysis is a crucial method to differentiate selegiline intake from drug abuse. A dried urine spot (DUS) analytical method based on spotting urine samples (10 μL) onto dried spot collection cards, and followed by air-drying and extraction, was developed and validated for the determination of selegiline, desmethylselegiline, R/S-methamphetamine, and R/S-amphetamine.

**Methods:** Methanol (0.5 mL) was found to be the ideal extraction solvent for target extraction from DUSs under orbital-horizontal stirring on a lateral shaker at 1,450 rpm for 30 min. Determinations were performed by direct electrospray ionization tandem mass spectrometry (ESI-MS/MS) under positive electrospray ionization conditions using multiple reaction monitoring mode. The chromatographic system consisted of a Chirobiotic^TM^ V2 column (2.1 × 250 mm, 5 μm) and a mobile phase of methanol containing 0.1% (v/v) glacial acetic acid and 0.02% (v/v) ammonium hydroxide.

**Results and conclusions:** The calibration curves were linear from 50 to 5,000 ng/mL, with *r* > 0.995 for all analytes, imprecisions ≤ 15% and accuracies between −11.4 and 11.7%. Extraction recoveries ranged from 48.6 to 105.4% with coefficients of variation (CV) ≤ 13.7%, and matrix effects ranged from 45.4 to 104.1% with CV ≤ 10.3%. The lower limit of quantification was 50 ng/mL for each analyte. The present method is simple, rapid (accomplished in 12 min), sensitive, and validated by a pharmacokinetic study in human urine collected after a single oral administration of SG.

## Introduction

Dried spot cards have been reported as an easy, simple, fast, and inexpensive sampling strategy for specimen collection, transport, and storage (Li and Tse, 2010; Déglon et al., 2012; Demirev, 2013; Zimmer et al., 2013). In recent years, the applications of dried spot cards have been extensively reported to include biomarker analysis and drug testing in blood (Barfield et al., 2008; Spooner et al., 2009; Michely et al., 2017; Namdev et al., 2018). Nevertheless, additional biological fluids can be treated in the same way. Urine is the most widely employed matrix among the different biological specimens for the analysis of drugs and metabolites because of ease of collection and higher levels of drugs and metabolites, in comparison to the values found in serum/blood and saliva (Moeller et al., 2008). Moreover, the application of dried urine spot (DUS) technology has increased gradually, mainly in clinical diagnostics and forensic toxicology analysis (Al-Dirbashi et al., 2011; Forman et al., 2012; Lee et al., 2013; Otero-Fernández et al., 2013).

Selegiline (R(-)-N-methyl-N-(1-phenyl-2-propyl)-2-propinylamine, l-deprenyl, SG), a selective and irreversible inhibitor of monoamine oxidase type B used as an antiparkinsonian agent, is mainly excreted in urine as desmethylselegiline (DM-SG), R-methamphetamine (R-MA) and R-amphetamine (R-AM) (Maurer and Kraemer, 1992). Hence, selegiline intake may result in positive test results for MA or AM and pose a challenge for the interpretation of AM and MA positive body fluid test results (Maurer and Kraemer, 1992; Kim et al., 2000; Liu et al., 2017). It is necessary to distinguish legitimate therapeutic medicinal use from drug abuse in forensic science.

To solve the problem, a chiral analysis method for simultaneous determination of SG, DM-SG, R/S-MA, and R/S-AM should be developed to distinguish SG intake from drug abuse. Numerous studies have addressed the enantiomeric separation of MA and/or AM in different matrices using GC-MS (Maurer and Kraemer, 1992; Mohr et al., 2012), LC-MS (Wang et al., 2018; Chen et al., 2019), CE (Sevcík et al., 1996; Kim et al., 2000; Mikuma et al., 2016), PS-MS (Yang et al., 2018), and molecularly imprinted sulfonic acid functionalized resin (Alatawi et al., 2018). Moreover, in these studies, urine has been the most extensively reported specimen to differentiate SG use from MA abuse. However, no methods have been published describing the simultaneous determination of SG, DM-SG, R/S-MA, and R/S-AM on DUS.

The objective of the current work was to develop a DUS methodology as a simple, low cost, and effective sample pretreatment method for the simultaneous determination of SG, DM-SG, R/S-MA, and R/S-AM urine specimens. The present method could help to distinguish SG intake from drug abuse. Moreover, the well-validated method was applied to the pharmacokinetic study in human urine collected after a single oral administration of SG. The pharmacokinetic study could help to could provide supplementary interpretation for urine tests in forensic science.

## Materials and Methods

### Materials and Reagents

Methanolic solutions (1 mg/mL free base) of pure S-MA, R-MA, S-AM, and R-AM were obtained from Cerilliant (Round Rock, TX, USA). SG and DM-SG were obtained from Toronto Research Chemicals Inc. (Toronto, Canada). The internal standard (IS) 4-phenylbutylamine was purchased from Tokyo Chemical Industry (Tokyo, Japan). HPLC-grade methanol was purchased from Sigma–Aldrich (St. Louis, MO, USA). Ammonium hydroxide solution (25%) was purchased from Aladdin Chemistry Co. Ltd. (Shanghai, China). Acetic acid (99.8%) was purchased from CNW Technologies GmbH (Dusseldorf, Germany). Deionized water purified with a Milli-Q system (Millipore, MA, USA) was used for preparing solutions. Whatman (GE Healthcare, Maidstone, U.K.) supplied the FTA^TM^ Classic Card used for DUSs.

### Instruments and Conditions

The chromatographic analysis was performed using an Acquity UPLC System (Milford, USA). The enantioselective separation was achieved using a Supelco Astec Chirobiotic^TM^ V2 column (2.1 × 250 mm, 5 μm) with vancomycin as a chiral stationary phase. The temperature of the column compartment was set to 25°C. It was an isocratic elution and the mobile phase consisted of methanol with 0.1% (v/v) glacial acetic acid and 0.02% (v/v) ammonium hydroxide. The flow rate was 0.33 mL/min. The injection volume was 5 μL, and the total run time was 12 min.

Mass spectrometric detection in positive ion multiple reaction mode (MRM) was performed using a QTRAP® 6500 triple quadrupole mass spectrometer (AB SCIEX, Inc., USA) with electrospray ionization. The source conditions were chosen to acquire a satisfactory signal for all analytes as follows: source temperature, 500°C; curtain gas, 40 psi; collision gas, medium; ion spray voltage, 5,500 V; ion source gas 1, 45 psi; ion source gas 2, 45 psi. The MS/MS parameters are shown in Table 1. The molecular structural and properties of the analytes are shown in Table S1.

**Table 1:** Liquid chromatography tandem-mass spectrometry parameters for six analytes (quantifier ion in bold).

**Analyst**	**Retention time (min)**	**Precursor ion (Q1, m/z)**	**Product ion (Q3, m/z)**	**Collision energy (V)**	**Declustering potential (V)**	**Entrance potential (V)**	**Cell exit potential l(V)**
SG	3.0	188	**91**	20	70	10	13
			119	20			
DM-SG	4.1	174	**119**	13	50	10	13
			91	24			
S-AM	5.5	136	**119**	11	50	10	13
			91	22			
R-AM	6.6	136	**119**	11	50	10	13
			91	22			
S-MA	7.4	150	**119**	14	50	10	13
			91	23			
R-MA	8.1	150	**119**	14	50	10	13
			91	23			
4-Phenylbutylamine(IS)	6.7	150	**91**	15	60	10	13
			133	5			

Data acquisition was performed with Analyst 1.6.3 software (AB SCIEX, Inc., USA), and the raw data were processed using MultiQuant 3.0.2 (AB SCIEX, Inc., USA).

Other laboratory devices employed included an Advanced Digital Multi-Tube Vortex Mixer from Troemner Company (Thorofare, NJ, USA) and a MiniSpin plus centrifuge from Eppendorf (Hamburg, Germany).

### Urine Specimens

Authentic urine samples were collected from the Academy of Forensic Science institutional review board (IRB)-approved study that included controlled SG administration (dose within the normal therapeutic margin) to eight healthy volunteers through written consent in accordance with the Declaration of Helsinki. Drug-free urine specimens were collected from laboratory personnel volunteers with no drug history. Each urine specimen was collected in clean, sealed polyethylene vials maintained frozen at −20°C until analysis.

### Preparation of Stock and Working Solutions

A mixed stock solution of SG, DM-SG, S-MA, R-MA, S-AM, and R-AM at concentrations of 1 μg/mL was prepared in methanol. Other standard solutions for the calibration and quality control (QC) samples were prepared daily by appropriate dilution with drug-free urine from the mixed solution. Calibration samples were made at concentrations of 50, 100, 250, 750, 1,250, 2,500, and 5,000 ng/mL. QC samples were made at 4 concentrations: lower limit of quantification (LLOQ), 50 ng/mL; low QC (LQC), 200 ng/mL; medium QC (MQC), 500 ng/mL; high QC (HQC), 4,500 ng/mL. Moreover, an IS stock solution (100 ng/mL) was prepared in methanol. All of the stock solutions were stored at −20°C until utilized.

### Sample Preparation

Each Whatman FTA^TM^ Classic Card was previously divided into 1.5 × 1.5 cm segments. Urine spots were then generated by dispensing 10 μL of samples (urine) into the center of the 2.25 cm^2^ card and allowing the spots to air dry for 2 h inside a clean fume hood.

DUS samples were extracted by manually punching a 10 mm disc from the center of the cards and inserting it into a 2 mL polypropylene microtube containing 10 μL of IS solution and 500 μL methanol. The microtube was capped and mixed for 30 min at 1,450 rpm on a lateral shaker. Then, the dried spot disc was removed and the microtube resealed. After centrifugation for 3 min at 12,000 rpm, 20 μL of the supernatant was diluted 4 times with 60 μL methanol. The mixture was transferred to the autosampler vial and injected into the LC-MS/MS system.

### Method Validation

The validation was performed in accordance with the guidelines provided by the US FDA for method validation (Food and Drug Administration, U.S. Department of Health and Human Services, 2001; Peters et al., 2017; Wille et al., 2017).

To investigate the method selectivity, six different blank DUS samples and zero samples (blank samples + IS) were tested for any interference peaks around the retention times of the analytes or IS. The sensitivity was assessed by establishing the limit of detection (LOD) and LLOQ for all components. LOD was calculated as at least 3 times of the signal to noise ratio (S/N). The LLOQ was expressed as the lowest concentration on the calibration curve which the S/N was at least 10. Calibration curves were established by preparing 7 non-zero standard samples (50, 100, 250, 750, 1,250, 2,500 and 5,000 ng/mL). Carryover was assessed by injecting two blank DUS samples after the highest calibration standard (5,000 ng/mL). For the analytes and IS, the carryover should have been <20 and 5% of the peak response of LLOQ samples within the same batch, respectively.

The precision was expressed as relative standard deviation (RSD%), and the accuracy was expressed as relative error (RE%). Intra-batch (*n* = 6) precision and accuracy for analytes throughout the calibration range for DUS sampling were evaluated by the analysis of QC samples at four different QC sample concentrations (LLOQ, LQC, MQC, HQC) as determined against a standard calibration curve in a single batch. Inter-batch precision and accuracy were evaluated similarly for the duplicate analysis of QC samples in four separate batches. Six lots of blank urine samples from six individuals were utilized to prepare the QC samples to assess the matrix effect (ME) and recovery. These experiments were performed with the LQC, MQC, and HQC. Sample set 1 represented the neat standard prepared in mobile phase, sample set 2 represented QC samples that were prepared by spiking blank matrix postextraction, and sample set 3 represented extracted DUS QC samples. For recovery determination, peak areas from sample set 3 were compared to those of sample set 2; for the ME, peak areas from sample set 2 were compared to those of sample set 1.

Processed sample stability was assessed to investigate the stability of analytes during sample collection. The set of LQC, MQC, and HQC stored at RT for 0 h was the reference group, and the other six sets, which were stored at RT for 4, 8, 12, 16, 20, and 24 h, were the test groups. Samples from the three sets of QCs were prepared and analyzed in six replicates in the same batch together with a calibration curve. If the recoveries of all seven analytes in a test group were within 85–115% compared with the reference group, and the CVs were <15%, the analytes were considered stable under the same condition as the test group.

### Pharmacokinetic Application

The validated analytical method was applied to urine samples stored as DUS from participants after a single 10 mg oral dose of SG. The eight healthy volunteers (four males, four females) were non-smokers and did not regularly use medications. The mean age of the participants was 27 ± 3 years (range, 23–31 years), the mean height was 170 ± 6 cm (range, 161–176 cm), and the mean weight was 58 ± 8 kg (range, 45–70 kg).

Urine specimens were collected from 0.5 h before and up to 168 h (7 d) after oral administration: in particular, samples were collected 0.5 h before drug administration and 0.5, 1.0, 1.5, 2.0, 2.5, 3.0, 4.0, 5.0, 6.0, 7.0, 9.0, 11.0, 13.0, 24.0, 28.0, 31.0, 48.0, 52.0, 55.0, 72.0, 79.0, 96.0, 103, 120, 127, 144, and 168 h after ingestion. Urine samples were transferred into 10 mL polypropylene tubes, capped and stored at −20°C. Prior to analysis, samples were thawed, and 10 μL was dispensed onto dried spot cards. The DUS samples were then dried for 2 h at room temperature in a clean fume hood. After that, the DUS samples were stored in sealed plastic bags at room temperature until analysis. Each urine sample was processed twice according to the aforementioned method.

The pharmacokinetics of SG, DM-SG, R-MA, and R-AM were evaluated using Excel 2016 (Microsoft Corporation). Pharmacokinetic analysis included determination of the following parameters: maximum concentration (C_max_) and time of maximum concentration (T_max_).

## Results

### Analytical Method Validation

**Figure 1:**
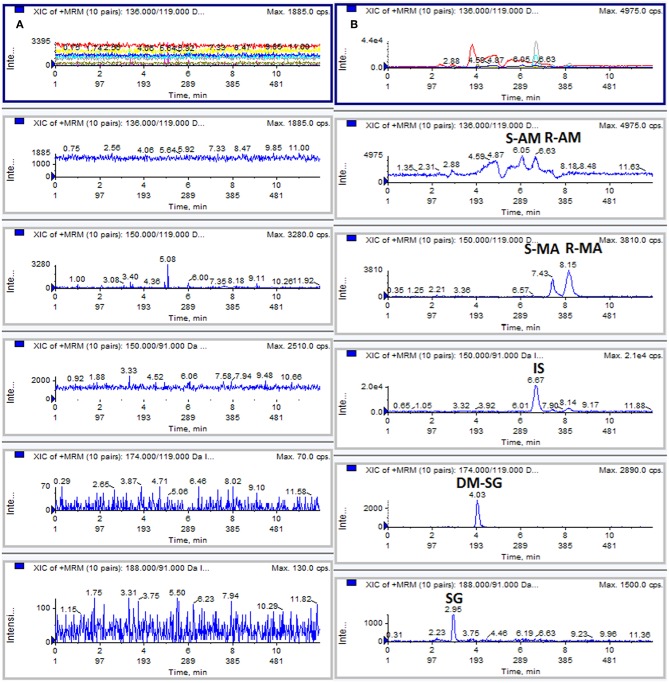
Typical MRM chromatograms: **(A)** blank DUS sample; **(B)** DUS sample spiked with analytes at the LLOQ level (50 ng/mL).

No significant interference was observed at the retention time of the analytes and IS, indicating acceptable method selectivity. Figure 1 shows the typical extracted ion chromatograms obtained from the analysis of a blank DUS sample and a drug-free DUS sample spiked at the LLOQ level (50 ng/mL). The LOD and LLOQ were 20 ng/mL and 50 ng/mL for each analyte.

A weighted factor of 1/x (inverse of the concentration) was used for all calibration curves. The calibration curve showed a linearity of analyte concentrations between 50 and 5,000 ng/mL with a correlation coefficient (*r*) > 0.995 (Table 2). The high *r* value indicated good linearity, and the standard deviation values indicated the significant validity of the calibration points. No carryover effect was observed in the blank DUS samples measured directly after the highest calibration standard. The results of within-batch and between-batch accuracy and precision are shown in Table 2. The acceptance criteria of accuracy and precision were met.

**Table 2:** Precision and accuracy.

**Analyst**	**Regression equation**	**Correlation coefficient (*r*)**	**Concentration spiked (ng/mL)**	**Intra-batch (*****n*** **=** **6)**			**Inter-batch (*****n*** **=** **24)**		
				**Concentration found (ng/mL)**	**Accuracy (%)**	**Precision (%)**	**Concentration found (ng/mL)**	**Accuracy (%)**	**Precision (%)**
SG	Y = 3.90478e^−4^x + 0.01041	0.9952	50^*^	51.5	3.0	9.9	41.3	−17.5	19.0
			200	193.7	−3.2	4.8	209.9	5.0	15.0
			500	499.0	−0.2	3.2	512.9	2.6	13.8
			4,500	4, 689.6	4.2	4.0	4, 804.1	6.8	13.5
DM-SG	Y = 0.00108x – 0.00456	0.9955	50^*^	41.2	−17.5	7.9	48.4	−3.2	16.7
			200	200.8	0.4	1.4	194.4	−2.8	14.6
			500	506.6	1.3	2.8	446.8	−10.6	14.3
			4,500	4, 596.0	2.1	3.7	4, 089.3	−9.1	14.7
S-AM	Y = 0.00166x + 0.01477	0.9979	50^*^	55.4	10.7	12.1	5.3	1.5	10.4
			200	209.1	4.5	2.8	11.8	6.3	5.5
			500	519.8	4.0	1.0	29.8	0.0	6.0
			4,500	4, 715.5	4.8	3.2	236.6	2.6	5.1
R-AM	Y = 0.00187x + 0.03629	0.9969	50^*^	47.5	−5.1	6.9	46.9	−6.2	14.9
			200	203.4	1.7	5.1	209.1	4.5	3.5
			500	504.6	0.9	2.8	516.3	3.3	4.0
			4500	4, 674.5	3.9	3.0	4, 558.3	1.3	5.1
S-MA	Y = 0.00151x + 0.02417	0.9954	50^*^	48.0	−4.1	9.9	44.3	−11.4	13.7
			200	202.0	1.0	2.2	207.1	3.5	10.9
			500	488.8	−2.2	4.7	493.8	−1.2	11.4
			4,500	4, 287.4	−4.7	8.5	4, 491.7	−0.2	8.8
R-MA	Y = 0.00370x + 0.03638	0.9958	50^*^	50.2	0.4	5.0	47.0	−6.0	6.8
			200	219.5	9.7	3.7	223.5	11.7	5.7
			500	529.6	5.9	4.0	527.9	5.6	3.4
			4,500	4, 610.6	2.5	2.2	4, 430.5	−1.5	2.9

* *Means LLOQ points*.

The MEs were 95.3–104.1% for SG, 95.4–101.5% for DM-SG, 88.8–94.2% for S-AM, 90.7–100.1% for R-AM, 45.6–61.9% for S-MA, and 85.7–91.8% for R-MA. Across the three concentration levels, the CVs were 1.2–2.5%, 0.3–1.7%, 1.2–2.9%, 1.2–2.9%, 5.9–10.3%, 1.6–3.6% for SG, DM-SG, S-AM, R-AM, S-MA, and R-MA. Thus, the MEs of analytes stayed constant across the three concentration levels. Only the MEs of S-MA were approximately 50%, which exhibited ion suppression with this method; the other analytes, SG, DM-SG, R-MA, S-AM, and R-AM, showed no significant MEs.

The recoveries were 48.6–68.0% for SG, 66.2–83.8% for DM-SG, 85.3–92.6% for S-AM, 90.8–105.4% for R-AM, 86.2–97.7% for S-MA, and 89.3–98.3% for R-MA. Across the three concentration levels, the CVs were 1.5–8.7%, 1.9–13.7%, 0.8–2.7%, 1.8–10.5%, 4.2–9.3%, 0.6–2.4% for SG, DM-SG, S-AM, R-AM, S-MA, and R-MA. Therefore, the recoveries of analytes are stable.

As for the process stability during sample collection, the recoveries of SG, DM-SG, S-AM, R-AM, S-MA, and R-MA were 106.5–109.6%, 90.7–102.2%, 95.7–103.4%, 92.4–100.7%, 91.5–113.3%, and 99.6–112.1% at RT for 24 h. Thus, they showed good stability during sample collection.

### Application of the Method

**Figure 2:**
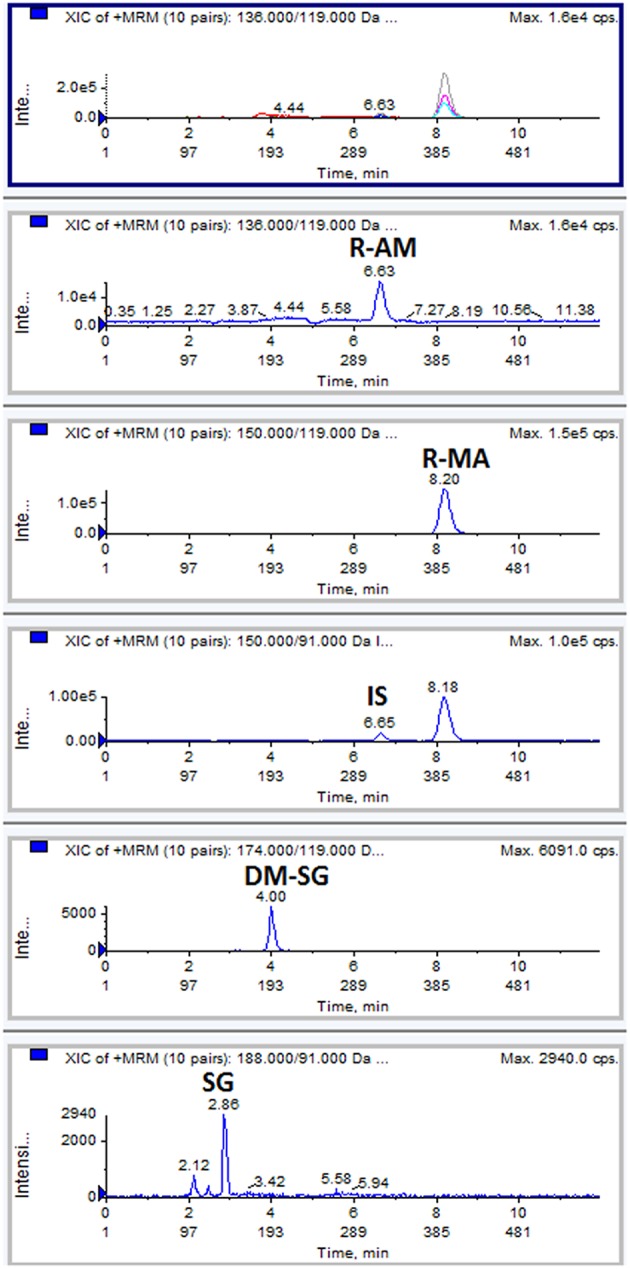
Typical MRM chromatograms: a DUS specimen collected at 1 h from one participant after the oral administration of 10 mg SG.

**Figure 3:**
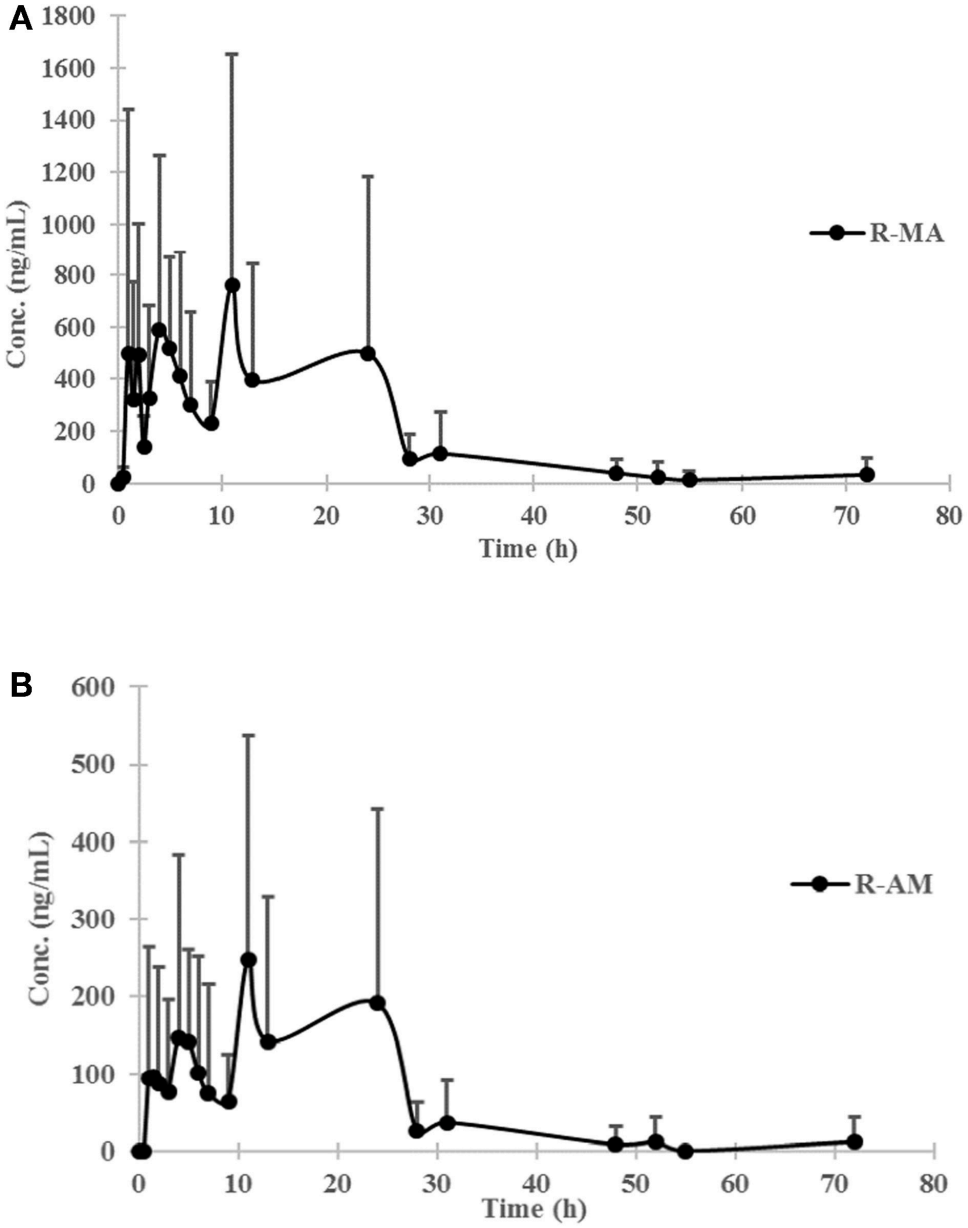
Urine concentration-time profiles of R-MA **(A)** and R-AM **(B)** in eight participants (four female, four male) receiving a single-dose administration of 10 mg of SG prepared by extraction from DUS. Error bars represent standard error of the mean.

The chromatogram of one specimen collected 1 h after the oral administration is shown in Figure 2. This sample contained 144.8 ng/mL SG, 153.6 ng/mL DM-SG, 2,777.0 ng/mL R-MA, and 417.1 ng/mL R-AM. The pharmacokinetic parameters C_max_, T_max_ for SG, DM-SG, R-MA, and R-AM in eight participants (four female, four male) receiving a single-dose administration of 10 mg of SG, prepared by extraction from DUS, are summarized in Table 3. The urine concentration-time profiles of R-MA and R-AM are presented in Figure 3. Considerable between-subject variability in C_max_, T_max_ values were apparent. R-MA and R-AM account for the major metabolites in urine. For SG, only one of the eight participants had a concentration over the LLOQ (50.0 ng/mL): 144.8 ng/mL 1 h after the oral administration. For DM-SG, only three of the eight participants had concentrations over the LLOQ (50.0 ng/mL) after the oral administration: the range of these values was 51.8–188.9 ng/mL. The times when the DM-SG was last detected in urine were 1–4 h.

**Table 3:** Mean analytes Cmax, Tmax in eight participants receiving a single-dose administration of 10 mg of SG prepared by extraction from DUS.

**Analytes**	Volunteers, n^*^	**C**_****max****_ **(ng/mL)**		**T**_****max****_ **(h)**	
		**Mean**	**Range**	**Mean**	**Range**
SG	1	144.8	144.8	1.0	1.0
DM-SG	3	162.5 ± 23.3	144.9–188.9	1.2 ± 0.8	0.5–2.0
R-MA	8	1, 135.6 ± 927.8	277.9–2,158.8	7.8 ± 7.1	1.0–24.0
R-AM	8	353.7 ± 263.3	146.8–731.5	13.8 ± 9.0	4.0–24.0

* *N means the number of participants who had concentrations over LLOQ*.

**Figure 4:**
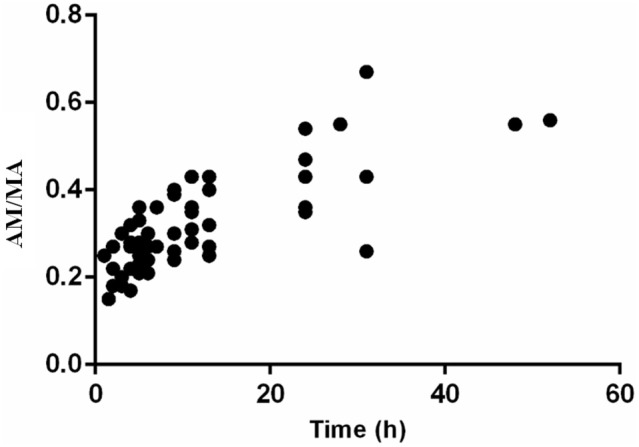
The ratios of AM to MA in urine after oral administration of 10 mg SG (*n* = 8).

The ratios of AM to MA (AM/MA) in urine ranged from 0.15 to 0.67 and maintained an upward trend along with time (*r* = 0.5865) after the SG administration (Figure 4).

In 2007, the US Substance Abuse and Mental Health Services Administration (SAMHSA) required the following cutoffs: MA for the confirmatory tests should be 500 ng/mL in urine containing AM at a concentration ≥200 ng/mL; AM for the confirmatory tests should be 500 ng/mL in urine (Bush, 2008). They also proposed in the same guideline that the cutoff concentration of MA should be set at 250 ng/mL in urine containing AM at a concentration ≥100 ng/mL and that the cutoff concentration of AM should be set at 250 ng/mL in urine (Bush, 2008). Mean detection times for the first and last positive DUS specimens confirmed by LC-MS/MS are presented in Table 4 using the set cutoff values. No analyte concentration values for the eight participants exceeded LLOQ 3 days after oral administration.

**Table 4:** Mean R-MA and R-AM detection times and ranges in eight participants receiving a single-dose administration of 10 mg of SG prepared by extraction from DUS.

**Analytes**	Cutoff^*^	Volunteers, n^**^	**Initial detection time (h)**		**Last detection time (h)**	
			**Mean**	**Range**	**Mean**	**Range**
R-MA	LLOQ	8	1.2 ± 0.5	0.5–2.0	39.8 ± 21.0	9.0–72.0
	250 ng/mL	7	6.3 ± 8.1	1.0–24.0	24.4 ± 6.0	13.0–31.0
	500 ng/mL	5	6.8 ± 9.7	1.0–24.0	15.4 ± 8.4	5.0–24.0
R-AM	LLOQ	8	2.8 ± 1.7	1.0–5.0	30.8 ± 18.8	5.0–72.0
	250 ng/mL	3	3.0 ± 2.6	1.0–6.0	20.3 ± 6.4	13.0–24.0
	500 ng/mL	3	9.3 ± 4.7	4.0–13.0	16.0 ± 7.0	11.0–24.0

* LLOQ, concentration of R-MA and R-AM at 50 ng/mL; 250 ng/mL, for methamphetamine, specimen must also contain amphetamine at a concentration ≥100 ng/mL; 500 ng/mL, for methamphetamine, specimen must also contain amphetamine at a concentration ≥200 ng/mL.

** *N means the number of participants who had concentrations over cutoff*.

## Discussion

### Analytical Method

An accurate, precise and selective method was developed to determine SG, DM-SG, R/S-MA, and R/S-AM on DUS. To our knowledge, no methods have been published describing the simultaneous determination of SG, DM-SG, R/S-MA, and R/S-AM on DUS. From a forensic analysis or clinical trial perspective, the developed DUS method presents the advantages of flexible sample collection and storage as well as reduced sample volumes. Aside from conventional validated methods, other factors had been reported to possibly affect the performance of dried spot card assays, mainly including IS addition, spot volume, hematocrit, spot homogeneity, and spot-to-spot carryover (Jager et al., 2014). The common practices reported for IS addition are to either add the IS to the extraction solvent and perform the other procedures such as pretreatment of the dried spot card with IS before spotting or to add the IS to the spotted sample (Meesters et al., 2011; Jager et al., 2014). The IS addition method in the present article adopted the former method, and the accuracy and precision were acceptable. Although spot volume was reported to exert minor effects on the analytical results by several articles (Vu et al., 2011; Jager et al., 2014; Gonzalez et al., 2015), Aida Serra has reported that increasing volumes of concentrated urine resulted in a reduction of the extraction recovery and an increase of the influence on the ionization caused by the matrix (Serra et al., 2013). In that article, four volumes (5, 10, 15, and 20 μL) were tested; the results met the criteria in terms of extraction recovery and ME obtained with 5 and 10 μL volumes. In the present article, we adopted 10 μL as the urine sample volume. Hematocrit and spot homogeneity are the factors most affecting the dried blood spots assay and were thus excluded from the discussion. In addition, spot-to-spot carryover can possibly be eliminated by punching blank DUS cards between punching DUS samples (Jager et al., 2014).

In 2007, SAMHSA required the initial and confirmatory cutoff concentrations for MA and AM of 1,000 and 500 ng/mL, respectively (Bush, 2008). In China, the Drug Concentration and Examination for Vehicle Drivers stipulates that the threshold concentrations of MA and AM in urine are each 1,000 ng/mL. In the present method, the LOD was 20 ng/mL and the LLOQ was 50 ng/mL for all analytes, which are sufficient to meet the criteria.

The difficulty in analyzing chiral drugs in biological matrices arises from the need to measure these compounds in low concentrations within high complexity matrices containing a diversity of low-molecular-mass compounds: other than the target analytes, salts, proteins, lipids, and complexes formed with metal ions are also present (Ribeiro et al., 2014). Sample preparation is a crucial step to successful and accurate quantification. In this study, dilution was optimized in terms of the DUS cleanup extraction solution. SG, DM-SG, R-MA, and R/S-AM showed no obvious MEs, whereas S-MA exhibited a remarkably different response. The MEs of S-MA were approximately 50%. Increased dilution can reduce the influence on the ionization caused by the matrix. In fact, when the DUS extraction solution was diluted 10-fold, the ME of S-MA could be up to 77.7% (the concentration of S-MA was 500 ng/mL; *n* = 3). Instead, the sensitivity would be reduced. In the present method, 4-fold dilution was adopted to meet the LOD of 20 ng/mL, as previously mentioned. In addition, since 4-phenylbutylamine was used as the IS, the ion-suppression effect did not significantly affect the accuracy of the results.

The reason that the recoveries of SG were comparatively low may be due to that the extraction solution is in direct contact with the surface of the spot and not in direct contact with the inner side. The migration of the extraction liquid at the inner side is rather slow and is characterized by diffusion (van der Heijden et al., 2009). Even though the recoveries of SG (48.6–68.0%) was comparatively low, the performance of the method was not adversely affected by the incomplete recovery, as demonstrated by the sufficient signal at the LLOQ and the validation of acceptable precision and accuracy.

### Pharmacokinetics of Selegiline in Urine

The mean R-MA, R-AM, and DM-SG Cmax values in urine reported in our study were 1,135.6 ± 927.8 (range 277.9–2,158.8 ng/mL), 353.7 ± 263.3 ng/mL (range 146.8–731.5 ng/mL), and 162.5 ± 23.3 ng/mL (range 144.9–188.9 ng/mL), respectively. In Francesco's reports, the Cmax values of R-MA and R-AM were approximately 150 ng/mL, and 50 ng/mL in oral fluid after a single oral administration of 10 mg SG (Jumex), respectively (Strano-Rossi et al., 2008). In plasma, the Cmax values of R-MA, R-AM, and DM-SG were 7.9–27.0 ng/mL (Heinonen et al., 1994; Kivistö et al., 2001), 14.0 ng/mL (Heinonen et al., 1994), and 11.3–19.5 ng/mL (Heinonen et al., 1994; Laine et al., 1999; Kivistö et al., 2001) with a comparable oral dose of 10 mg of SG. Higher Cmax values for R-MA, R-AM, and DM-SG were observed in urine than in plasma and oral fluid. Raf J.F. Schepers previously reported the same phenomenon that MA and AM concentrations in urine were considerably higher than the corresponding plasma and oral fluid concentrations after MA administration, facilitating prolonged drug detection (Oyler et al., 2002; Schepers et al., 2003).

The urinary R-MA concentrations exceeded 1,000 ng/mL in three of eight subjects after a single oral administration of 10 mg SG, which is the most common cut-off value used in the screening test for illicit MA use. In Jonathan M. Oyler's report, the latest detection times for MA after a single oral administration of 10 mg MA were 25–77 h based on the proposed cutoff values of 250 ng/mL (specimen must also contain AM at a concentration ≥100 ng/mL); the latest detection times for MA were 22–66 h based on the confirmatory cutoff values of 500 ng/mL (specimen must also contain AM at a concentration ≥200 ng/mL). Comparatively, these results overlapped partially with the last detection times in our report, which were 13–31 and 5.0–24 h after a single oral administration of 10 mg SG separately based on the cutoff values. This phenomenon undoubtedly increased the difficulty in differentiating SG therapy from illicit MA use.

Furthermore, urinary AM/MA has been reported as a possible distinguishable marker for selegiline use from other drugs, mainly because of the difference in values between SG users and MA users (Kim et al., 2000). In their article, AM/MA ratios for urinary concentrations after SG administration ranged from 0.28 to 0.36, whereas the urinary AM/MA ratios from MA abusers ranged from 0.04 to 0.37 (Kim et al., 2000). However, according to our results, the ratios of AM to MA ranged from 0.15 to 0.67, similar to a report in which the ratios of AM to MA ranged from 0.24 to 0.67 (Hasegawa et al., 1999). In addition, in Jonathan M. Oyler's report, the AM-MA ratios ranged from 0.25 to 2.6 in the last positive urine specimen after consecutive doses of MA (Oyler et al., 2002). Thus, the AM/MA ratios could not represent a sufficient distinguishable marker between SG therapy and illicit MA use.

The parent drug, SG, could only be detected in one participant's urine 1 h after oral administration. Although SG was undetectable in urine, according to several articles (Heinonen et al., 1994; Hasegawa et al., 1999), Ho-Sang Shin has reported the existence of SG in urine after its administration (Shin, 1997). In distinguishing SG therapy from illicit MA use, the detection of DM-SG, which is a specific metabolite of selegiline, was not always a conclusive marker. DM-SG rapidly disappeared from urine and cannot be detected in urine 5 h after oral administration, according to our results. Thus, the present chiral analysis of MA and AM would be a useful technique in forensic practice.

## Conclusions

A simple and rapid LC-MS/MS method for determination of SG, DM-SG, R/S-MA, R/S-AM on DUS has been developed and fully validated. Its application was demonstrated by the pharmacokinetics of SG, R-MA, R-AM, and DM-SG in urine after a single oral administration of 10 mg SG. The C_max_ values of R-MA, R-AM, and DM-SG in urine were higher than those reported in plasma and oral fluid. The ratios of AM to MA in urine ranged from 0.15 to 0.67 after SG administration and could not serve as a sufficient distinguishable marker between SG therapy and illicit MA use. No concentration values of any analyte exceeded LLOQ 3 days after oral administration. The present method can provide rapid and simple discrimination between SG use and illegal abuse. Furthermore, because of the simplicity of the method, the developed method is an appealing methodology for routine (screening and quantitative confirmation) analysis and efficient for the analysis of large numbers of samples for forensic and clinical application. In addition, the pharmacokinetic results could provide supplementary interpretation for urine tests in forensic science and drug treatment programs.

## Ethics Statement

Authentic urine samples were collected from the Academy of Forensic Science institutional review board (IRB)-approved study that included controlled selegiline administration (dose within the normal therapeutic margin) to eight healthy volunteers through written consent in accordance with the Declaration of Helsinki.

## Author Contributions

LC finished the experiments and data analysis and prepared the manuscript. YY, GD, XW, and BS assisted with experiments. PX conceived the experiments and revised the manuscript.

### Conflict of Interest Statement

The authors declare that the research was conducted in the absence of any commercial or financial relationships that could be construed as a potential conflict of interest.
