# Supplementary material for: Simultaneous Determination of Selegiline, Desmethylselegiline, R/S-methamphetamine, and R/S-amphetamine on Dried Urine Spots by LC/MS/MS: Application to a Pharmacokinetic Study in Urine
Source: Front Chem. 2019 Apr 17;7:248. doi: 10.3389/fchem.2019.00248 (PMC6478707; doi:10.3389/fchem.2019.00248)
Supplement: Supplementary file 1 [file Table_1.DOCX]

**Table S1** The molecular structural and properties of the analytes

| Analyst | molecular mass | pKa | LogP | molecular structural |
| --- | --- | --- | --- | --- |
| SG | 187.3 | 8.7 | 2.7 | 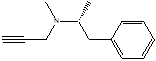 |
| DM-SG | 173.3 | 8.7 | 2.4 | 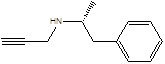 |
| S-AM | 135.2 | 10.0 | 1.8 | 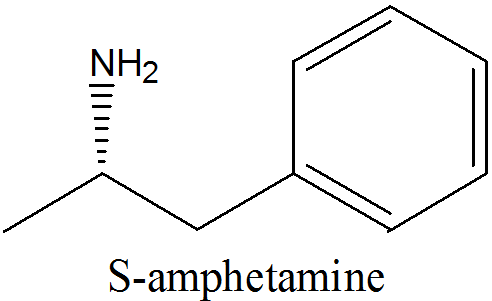 |
| R-AM | 135.2 | 10.0 | 1.8 | 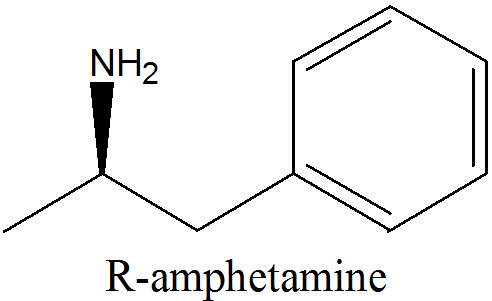 |
| S-MA | 149.2 | 10.2 | 2.2 | 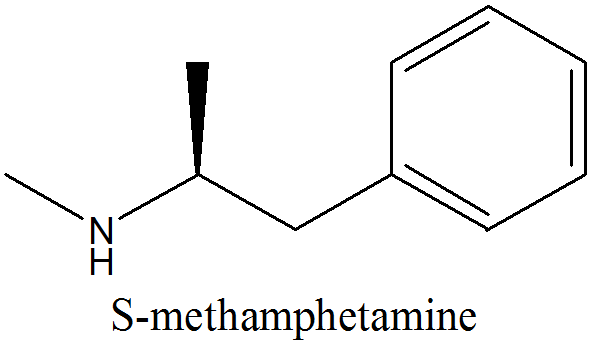 |
| R-MA | 149.2 | 10.2 | 2.2 | 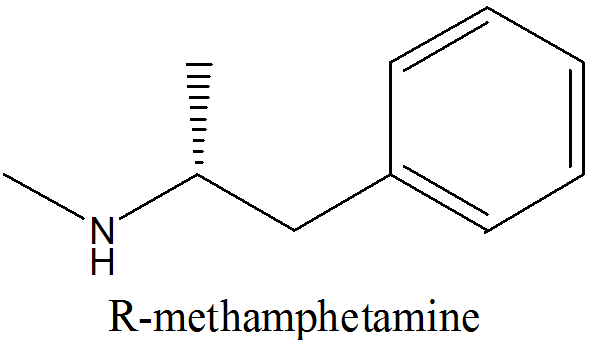 |
